# Supplementary material for: Efficient combinatorial optimization by quantum-inspired parallel annealing in analogue memristor crossbar
Source: Nat Commun. 2023 Sep 22;14:5927. doi: 10.1038/s41467-023-41647-2 (PMC10516914; doi:10.1038/s41467-023-41647-2)
Supplement: Supplementary file 1 — Supplementary Information [file 41467_2023_41647_MOESM1_ESM.pdf]

Efficient combinatorial optimization by quantum-inspired parallel annealing in analogue memristor crossbar

Mingrui Jiang, Keyi Shan, Chengping He and Can Li*

Department of Electrical and Electronic Engineering, The University of Hong Kong, Hong Kong SAR

*Email: canl@hku.hk

Contents

1 Supplementary Figures **2**

2 Supplementary Table **19**

3 Supplementary Note **20**

4 Supplementary References **21**

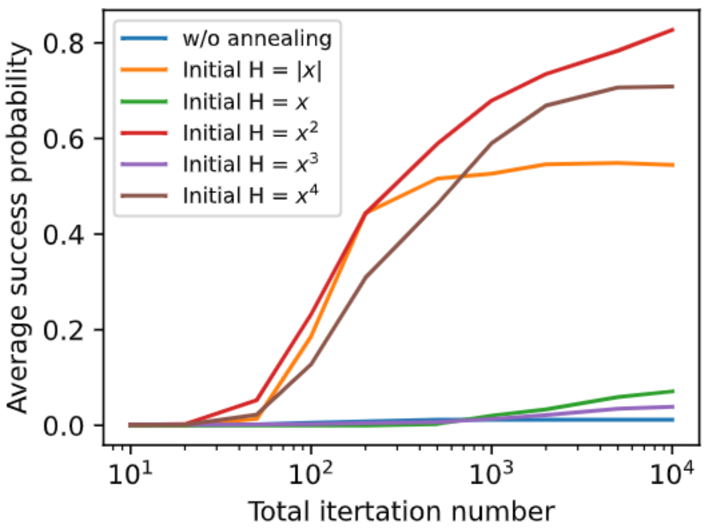

Figure S1. The impact of different initial Hamiltonians. This plot shows the average success probability of different $H_{initial}$ functions with different total iteration numbers. The curves with different color represent the case without $H_{initial}$, $H_{initial} = \sum_{i=1}^{N}|x_i|$, $H_{initial} = \sum_{i=1}^{N} x_i$, $H_{initial} = \sum_{i=1}^{N} x_i^2$, $H_{initial} = \sum_{i=1}^{N} x_i^3$ and $H_{initial} = \sum_{i=1}^{N} x_i^4$, respectively. For each data point, 20 60-node 50%-density unweighted Max-Cut problem instances are solved. For each problem instance, 100 trials from random initial states are conducted for calculating the success probability. The data in the plot shows the average success probability over 20 problem instances. From the plot, it can be observed that symmetric functions including $\sum_{i=1}^{N}|x_i|$, $\sum_{i=1}^{N} x_i^2$, $\sum_{i=1}^{N} x_i^4$, perform better than asymmetric ones. Since they all have an easily found ground state obtained when all $x_i = 0$. The success implementation of $\sum_{i=1}^{N}|x_i|$, $\sum_{i=1}^{N} x_i^2$ and $\sum_{i=1}^{N} x_i^4$ also implies that an arbitrary function can service as the initial Hamiltonian as long as its ground state can be easily solved. According to our empirical observation, among these three functions, $\sum_{i=1}^{N} x_i^2$ works the best, so it is chosen as the $H_{initial}$ throughout the paper. Nonetheless, it is possible that there are other well-designed functions that can enhance performance, which requires further exploration.

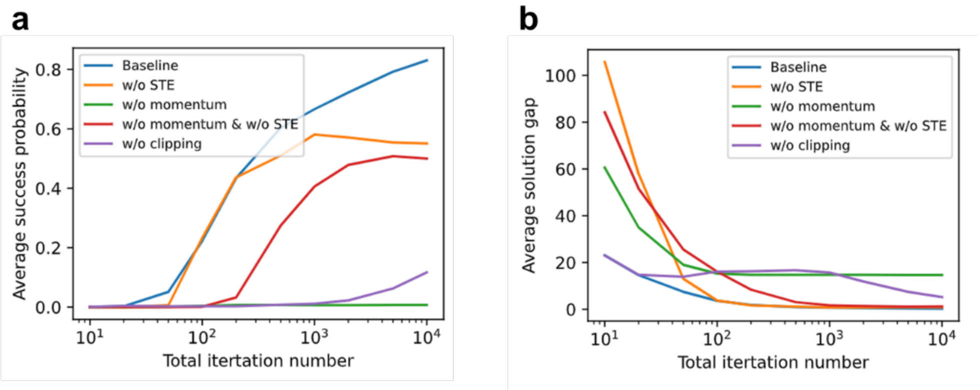

Figure S2. Comparison of different updating techniques in unweighted Max-Cut problem. a Average success probability vs. total iteration number. **b** Average solution gap (defined as the difference between the solved cut value and the maximum cut value) vs. total iteration number. Five different updating techniques are compared: the original updating algorithm used in the paper (denoted as Baseline in the figure), one without discretizing the proxy spin value when calculating the gradient (STE), one without adding a momentum term, one without momentum and without STE, and one without clipping the proxy spin value to be between -1 and 1. For each data point in the plot, 20 60-node 50%-density unweighted Max-Cut problem instances are solved. For each problem instance, 100 trials from random initial states are conducted. From the plot, it can be seen that all three techniques, STE, momentum, and clipping, are essential to the performance.

The performance of the case without STE works as well as with STE when the total iteration number is small. However, with larger total iterations, the performance decreases. This is because without STE, during the whole solving process, the real binary spin is not involved, and the analog spin proxy cannot accurately reflect the result of the binary spin, which causes a mismatch [1]. Further updating on the analog spin proxy lacks the ability to lower the energy calculated by the binary spin. For the case without momentum, the system works like a greedy gradient descent algorithm, and the annealing fails. For this case, a local-minimum solution can be easily obtained, however, the global minimum can hardly be found. The role of momentum in the quantum-inspired annealing is not clear yet, but this might be related to the property of STE. Since the performance of vanilla stochastic gradient descent is much poorer than those ones with momentum in the field of binary neural network (BiNN) training [2], similar with our observations. For the case without momentum and without STE, the annealing backs to work normally. The convergence speed is relatively slower than the case without momentum. For the case without momentum and without STE, the annealing works normally, but the convergence speed is relatively slower than the case without momentum. For the case without clipping, the analog spin proxy does not have any limitation, and it can grow infinitely towards one direction in the solving process. Then if it needs to go towards the other direction in the later solving process, it takes much longer time to accumulate the gradient until it can change its sign and thus largely decreases the solving speed. For STE, momentum, and clipping, they are all common techniques used in BiNN training. In fact, there are more advanced optimizers, such as Adam, and other sophisticated training methods that are widely applied in the field of neural network [3,4]. These techniques have the potential to be applied to combinatorial optimization, but their applicability needs to be further researched due to the different nature of the target problem.

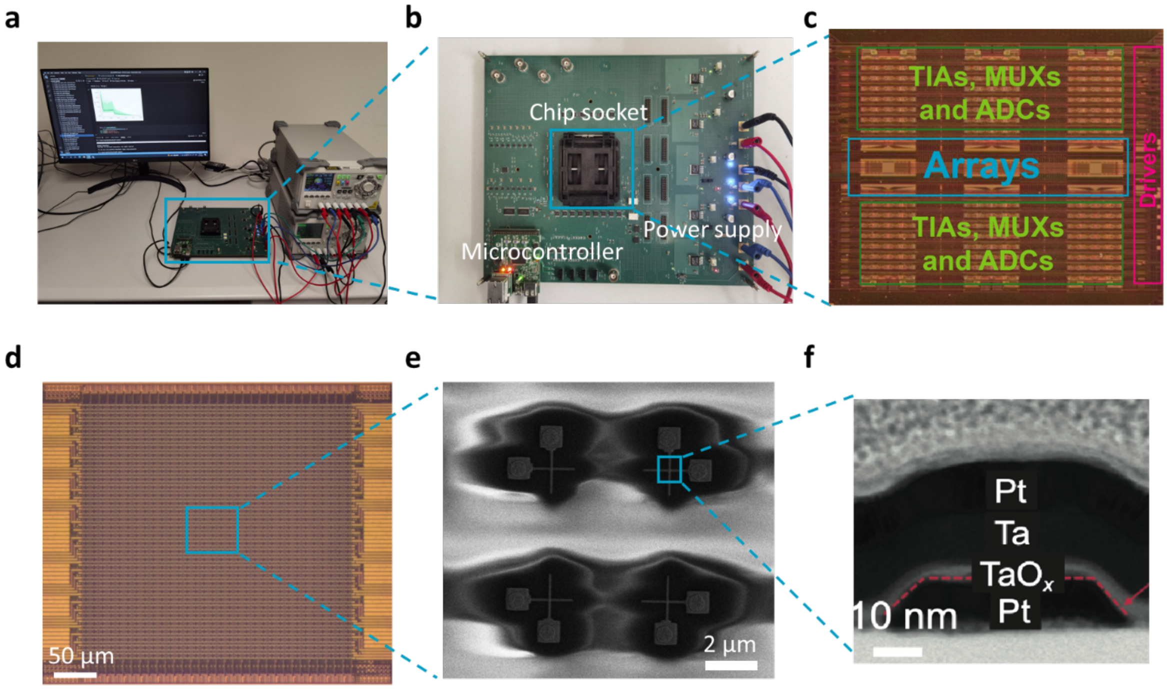

Figure S3. Experiment set-up and integrated memristor chip. a The overall experiment set-up. A PC is used to control the integrated memristor chip via microcontroller. **b** The printed circuit board (PCB) developed to measure the chip. **c** Optical image of the integrated memristor chip, which consists of three 64×64 1T1M memristor arrays and peripheral circuits including transimpedance amplifiers (TIAs), multiplexers (MUXs), analog-to-digital converters (ADCs) and drivers. CMOS peripheral circuits were taped out by TSMC's 180 nm technology, while the memristor devices were fabricated at backend of line with in-house cleanroom. **d** Optical image of the memristor crossbar array. **e** SEM image of the cross-point memristor device **f** Lateral TEM image of the 50 nm×50 nm Pt/Ta/TaO_x/Pt memristor device.

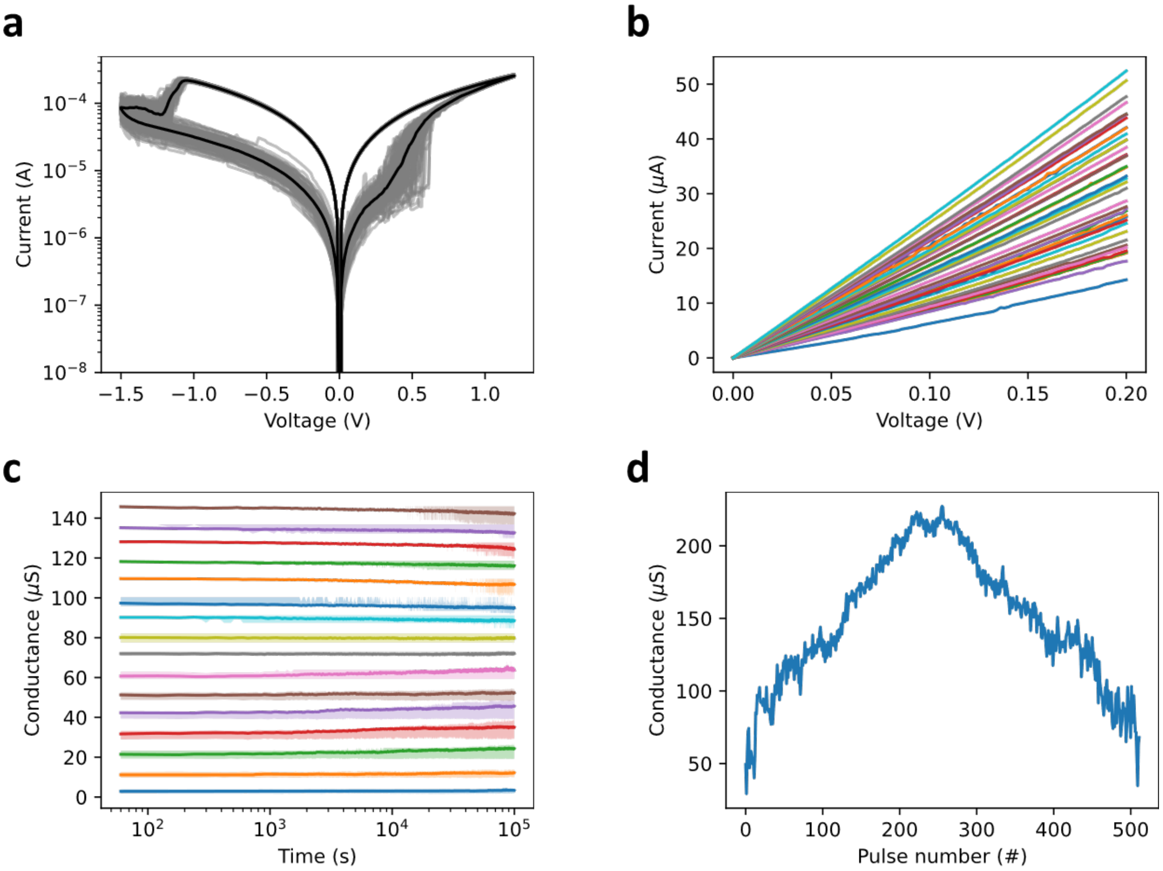

Figure S4. Performance of memristor device in our integrated hardware. a DC I-V measurement of 100 Set and Reset cycles demonstrating small cycle-to-cycle variations. The average value is shown by the dark line. **b** Linearity test of different conductance states, demonstrating excellent I-V linearity. **c** Retention test after dividing the entire 64×64 array into 16 subarrays, with each subarray programmed to target a conductance of 0 to 150 µS with a step size of 10 µS. The mean value is shown by the dark line, while the shaded area indicates the interquartile range. **d** Conductance tuning by a set of SET and RESET pulses. 256 SET pulses with amplitude linearly increasing from 0.6 V to 0.7 V were applied to the device followed by 256 RESET pulses with amplitude linearly decreasing from -0.5 V to -0.65 V. The pulse width for both SET and RESET pulses is 10 ns.

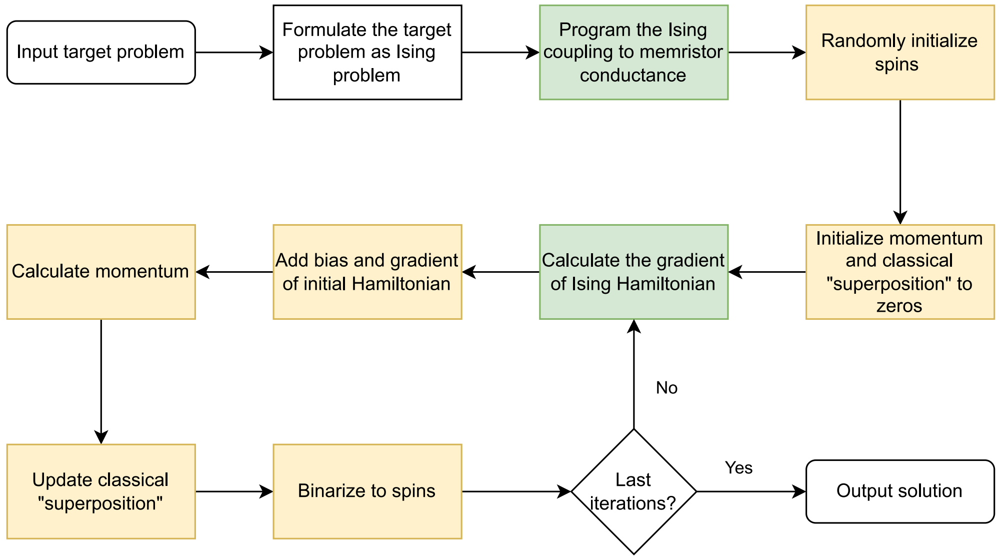

Figure S5. Flow chart of the proposed memristor-based Ising solver. Steps in green boxes were conducted experimentally in the analogue memristor crossbar array, while steps in yellow boxes were computing steps in the digital domain, which can be integrated onto the chip in the future.

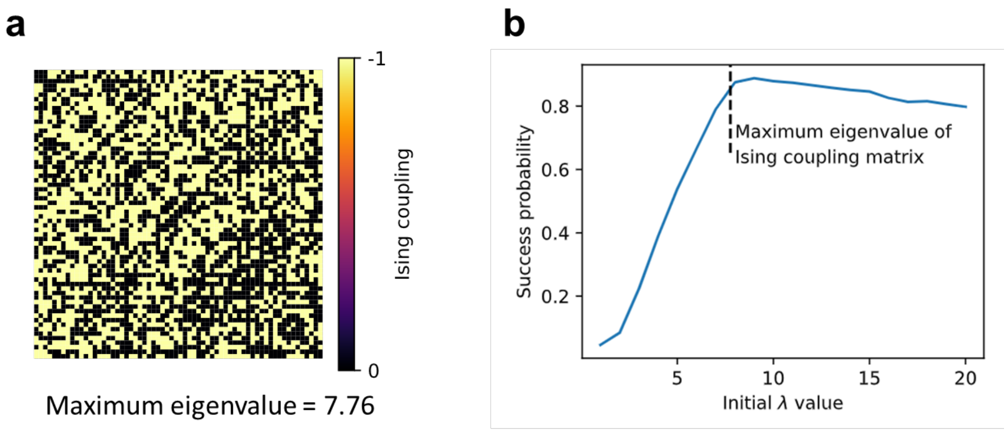

Figure S6. The impact of initial λ value and its relationship to maximum eigenvalue of Ising coupling matrix. a Ising coupling matrix. The maximum eigenvalue is 7.76. **b** Success probability of the problem instance in (a) vs. initial λ value. The black dash line shows the position of initial λ value equal to the maximum eigenvalue of Ising coupling matrix.

In our QPA, the coefficient of the initial Hamiltonian should start with a large enough that the system can be initiated with a function that the ground state can be easily found, i.e. usually a convex function. Since in our updating scheme, the gradient calculated by the binary spin σ_i is used as an estimator to update the analog proxy spin x_i. By replacing binary spin configuration $\boldsymbol{\sigma}$ in the Ising Hamiltonian with analog spin configuration $\boldsymbol{x}$, we have:

$$H_{system} = \frac{1}{2}\lambda\sum_i x_i^2 - \frac{1}{2}\sum_{i,j} J_{ij}x_ix_j = \frac{1}{2}(\lambda\boldsymbol{x}^T\boldsymbol{x} - \boldsymbol{x}^T\boldsymbol{J}\boldsymbol{x}) = \frac{1}{2}\boldsymbol{x}^T(\lambda\boldsymbol{I} - \boldsymbol{J})\boldsymbol{x}$$

In this case, if $\lambda\boldsymbol{I} - \boldsymbol{J} \geq \boldsymbol{0}$, the H_{system} becomes convex and has a ground state when all $x_i = 0$. Therefore, to guarantee a convex function, λ should start with a value that is larger than the maximum eigenvalue of Ising coupling matrix $\boldsymbol{J}$. However, it cannot be too large, as with further larger initial λ, more iterations will be wasted and thus affect the performance. From the above plot, it can be seen that the best performance point locates where initial λ value is a bit larger than the maximum eigenvalue of Ising coupling matrix, which further prove our theory.

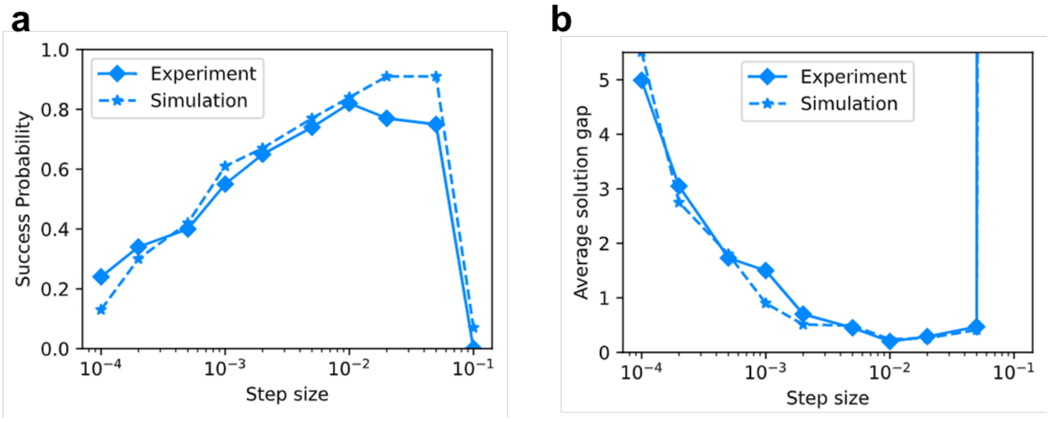

Figure S7. The impact of step size. a Success probability and **b** average solution gap vs. step size. For this plot, the target problem of Fig. 2c was solved with 100 trials. The dash line shows the simulation result with our experimentally validated memristor model, which complies well with the experimental data. From the plot it can be seen that, when the step size is relatively small, increasing it can lead to improved system performance due to each iteration progressing further in the direction of gradient. However, as the step size becomes sufficiently large, the system will diverge. This aligns with the experience in neural network training. In fact, more advanced adaptive changing of step sizes is commonly implemented in the training of neural networks [3,5,6]. The selection of optimal hyperparameters including step size, the coefficient of momentum term, is a vast and intricate problem that requires further research in the future.

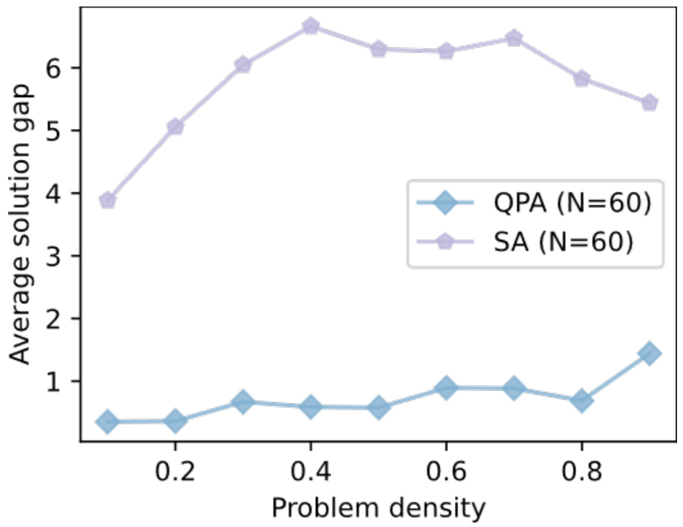

Figure S8. Comparison of average solution gap (optimal maximum cut - obtained cut) of our QPA and SA. Experiment condition of this plot is exactly the same as results shown in Fig. 2g in the main text.

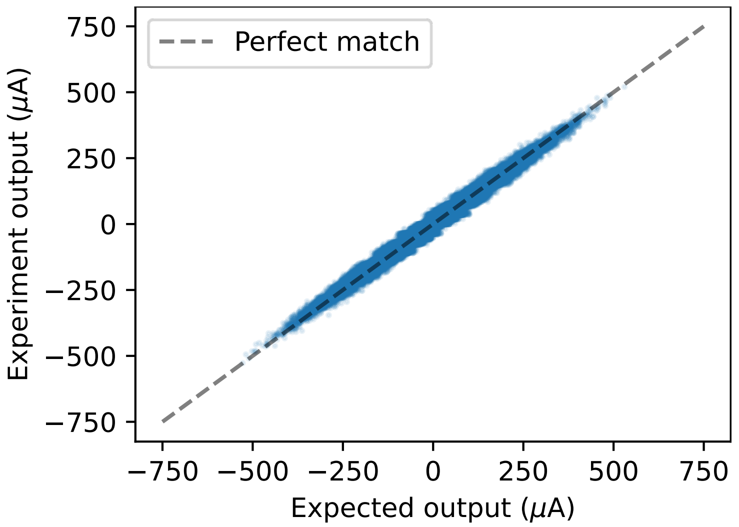

Figure S9. Experimental vector matrix multiplication result v.s. software calculated expected result. The gray dash line shows the line that experimental result perfectly matches the expected result.

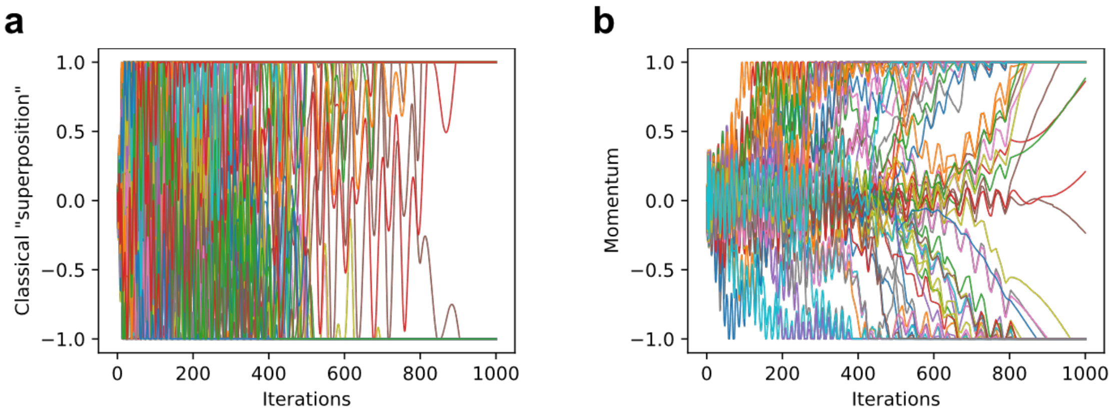

Figure S10. The evolution of intermediate values in experimental solving process of weighted Max-Cut problem. (a) The evolving process of classical "superposition" (analog spin proxy). (b) The evolving process of momentum. During the early stage of the solving process, the classical "superposition" rapidly oscillates between -1 and 1 due to higher impact of initial Hamiltonian, and eventually converges to -1 or 1 to match the binary spin configuration.

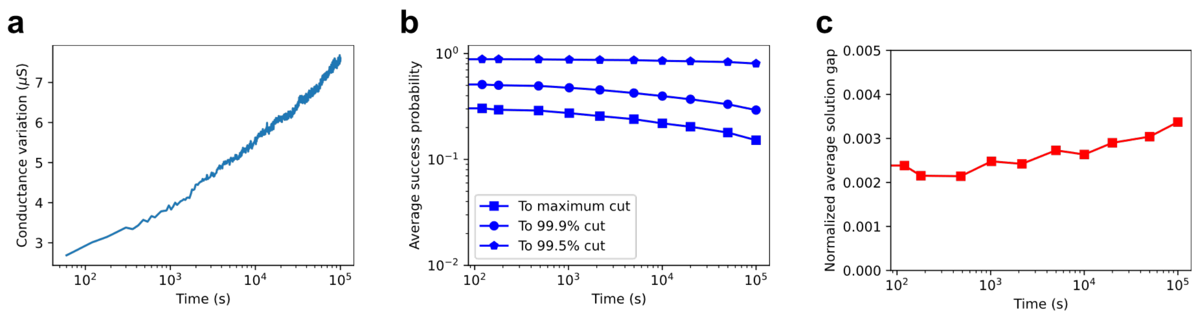

Figure S11. Exploration of the effect of conductance relaxation on the performance by simulation with the experiment-extracted model. (a) Experimentally measured relationship between the standard deviation of the conductance variation of the whole array and time. The measuring condition is the same as Fig. S4(c). With the increasing of retention time, the conductance variation of the memristor device gradually grows. (b) Change of average success probability with regard to the retention time. (c) Change of normalized average solution gap with regard to the retention time. For (b) and (c), the result is obtained by simulation with the experimentally measured conductance variation in (a). It can be seen that though with a long power-down period, the non-volatile property of memristor enables our system to get acceptable solutions with limited performance degradation.

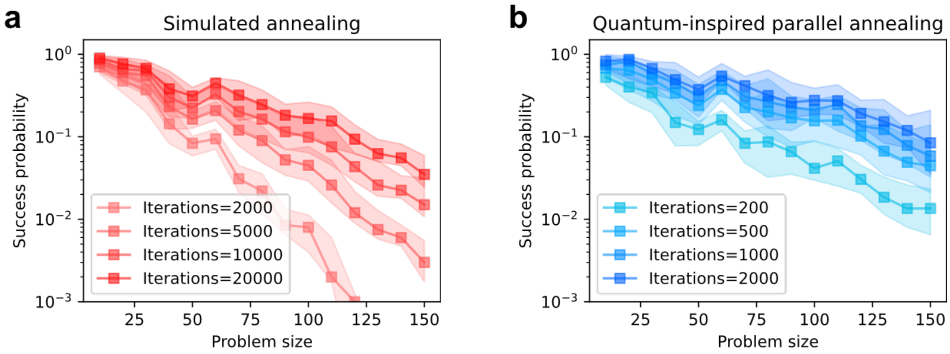

Figure S12. Success probability plot used for calculating TTS of weighted Max-Cut problem. a Success probability vs. problem size for simulated annealing **b** Success probability vs. problem size for quantum-inspired annealing. The result for quantum-inspired parallel annealing is obtained by simulation with experiment-validated memristor model.

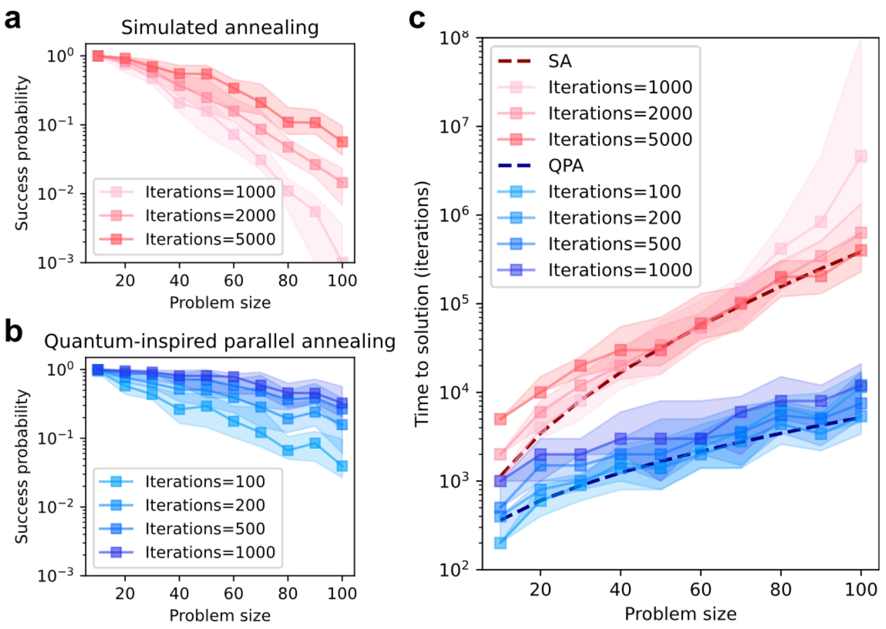

Figure S13. Scaling trend for unweighted Max-Cut problem. a Success probability vs. problem size for simulated annealing **b** Success probability vs. problem size for quantum-inspired annealing. **c** Time to solution vs. problem size. For each problem size, 20 randomly generated problem instances solved 1000 times with random initial spin configuration. The shaded area in the plot shows interquartile range. The square marker indicates the median. The result for quantum-inspired parallel annealing is obtained by simulation with experiment-validated memristor model.

a

Instance	Node	Edge	Density	Iterations	Best known cut	Obtained best cut	Difference	Obtained average cut	Success probability
G1	800	19176	0.06	10000	11624	11624	0	11609.59	0.161
G2	800	19176	0.06	10000	11620	11620	0	11605.19	0.05
G3	800	19176	0.06	10000	11622	11622	0	11612.83	0.306
G4	800	19176	0.06	10000	11646	11646	0	11633.02	0.056
G5	800	19176	0.06	10000	11631	11631	0	11621.21	0.325
G22	2000	19990	0.01	20000	13359	13359	0	13354.83	0.007
G23	2000	19990	0.01	20000	13344	13342	2	13335.48	(0.037)
G24	2000	19990	0.01	20000	13347	13347	0	13332.21	0.003
G25	2000	19990	0.01	20000	13340	13340	0	13327.84	0.002
G26	2000	19990	0.01	20000	13328	13328	0	13317.98	0.004

b

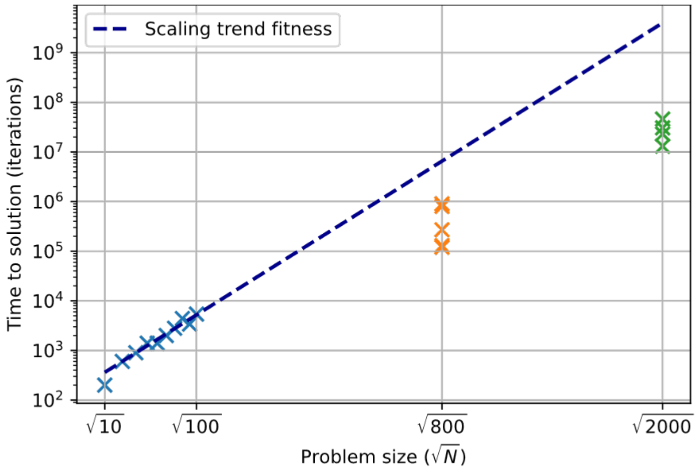

Figure S14. Verification of the scaling trend for larger problems with defect-free simulation. (a) The result of solving N=800 and N=2000 problems with random graphs from a MAX-CUT benchmark dataset, Gset (https://web.stanford.edu/~yyye/yyye/Gset/). For each problem instance, 1000 trials are conducted. The number in the bracket denotes the success probability of finding the obtained best cut. (b) Further scaling of the trend in Fig. S12(c) to N=2000. One major challenge for benchmarking the performance of large-size problems is to find exact solutions for them. As brute-of-force search becomes impossible for problems with N>60 even with the most advanced digital hardware due to the exponential explosion nature of the problem. Thus, Gset problems drawn from a public problem set are used for benchmarking. It can be seen that the scaling trend of solving larger problems is basically consistent with the fitness curve found in Fig. S12(c) with some deviations. There are two major reasons for the deviation. Firstly, problem instances in the Gset are much sparser than problems we solved in Fig. S12 (density=0.06 for N=800, density=0.01 for N=2000 compared with density=0.5 for N=10-100) and are generally easier to be solved according to our finding in Fig. 2(g) of the main text. Secondly, the simulation conducted in Fig. S12 considered the variation of the memristor device, while the simulation here is defect-free, which results in higher performance.

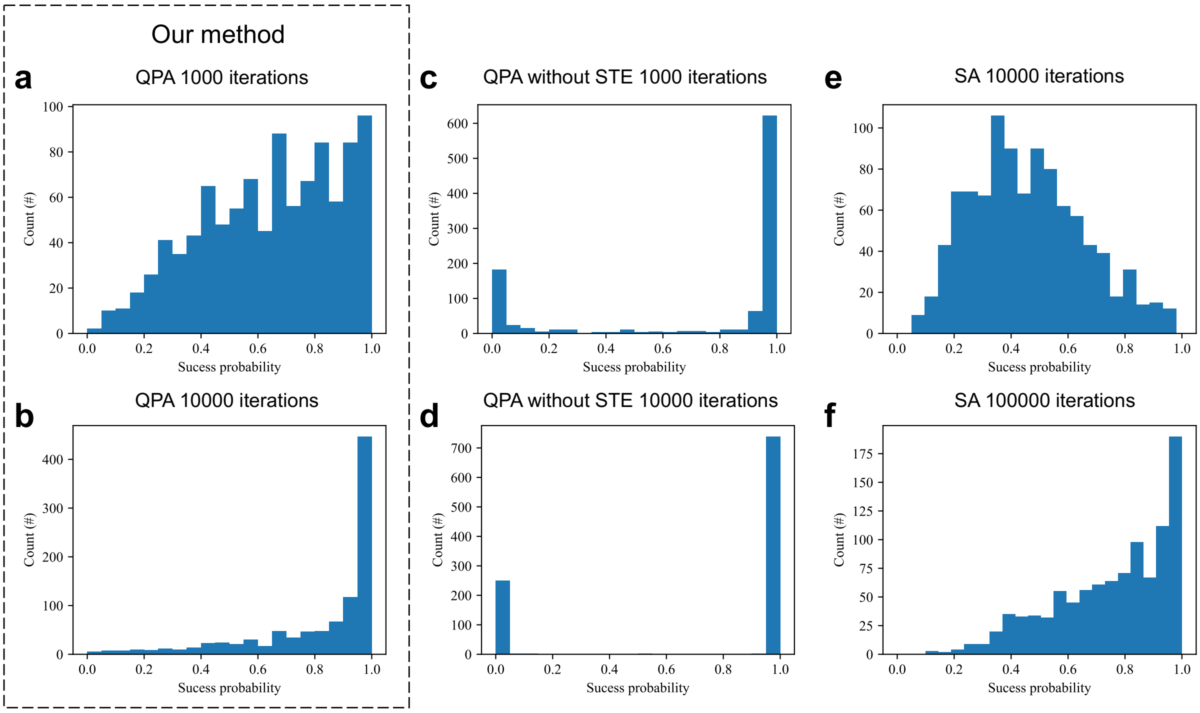

Figure S15. **Exploration of the effect of the hardness of the problem with defect-free simulation.** The success probability distribution of 1000 50%-density unweighted Max-Cut problem instances solved by (a)-(b) Original QPA with (a) 1000 iterations and (b) 10000 iterations. (c)-(d) QPA without discretizing the proxy spin value when calculating the gradient with (d) 1000 iterations and (e) 10000 iterations. (e)-(f) SA with (e) 10000 iterations and (f) 100000 iterations. For each problem instance, 100 random initialized trials are conducted to calculate the success probability. It can be seen that the histogram for QPA without STE is bimodal, meaning that it separates the problem instances into two groups, hard ones and easy ones. The algorithm almost always succeeds on easy problems and fails on hard problems. This kind of bimodal distribution is similar to the result obtained by the D-Wave quantum system and classical dynamics [7]. However, for original QPA and SA, the distribution is relatively unimodal. Further increasing of the total iteration number drives the distribution of QPA without STE more bimodal (The number of both easy and hard problems increases, while the number of problems in the middle decreases) but does not significantly improve the overall success probability. However, for both original QPA and SA, increased iterations cause the unimodal distribution to shift right, which stands for overall improved performance. The detailed mechanism behind the clear separation of hard and easy problems remains unclear. Here, we have a preliminary intuitive explanation. For QPA without STE, the whole solving process is based on the evolution of the analog spin value, meaning that the system tends to evolve to the ground state in the "analog" solution space, which might deviate from the true ground state in the binary solution space for some problem instances. Increasing iterations increases the ability to find the ground state in the "analog" solution space but does not help with finding the true ground state of binary spin. However, the introduction of STE in our QPA utilizes the binary spin value for the gradient calculation, which involves the true binary spin in the solving process and thus corrects the mismatch caused by the analog variable and leads to the true ground state. This might also explain why our method behaves better than those based on continuous-time dynamics such as optical and electronic oscillator-based systems.

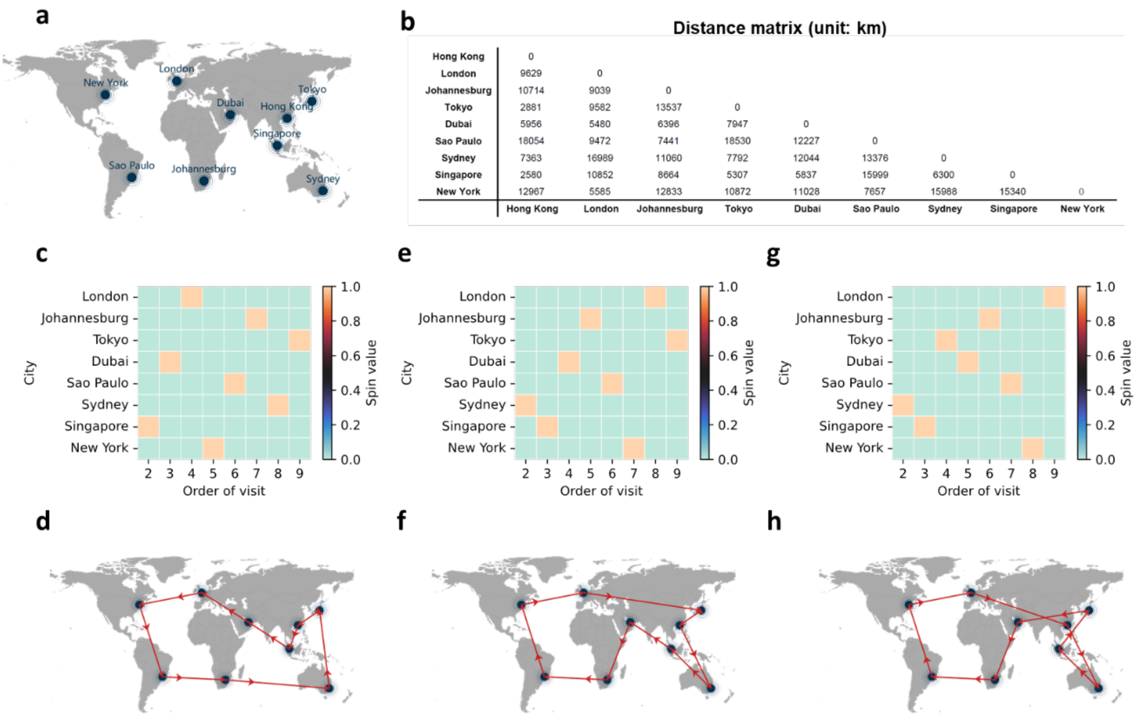

Distance matrix (unit: km)

	Hong Kong	London	Johannesburg	Tokyo	Dubai	Sao Paulo	Sydney	Singapore	New York
Hong Kong	0								
London	9629	0							
Johannesburg	10714	9039	0						
Tokyo	2881	9582	13537	0					
Dubai	5956	5480	6396	7947	0				
Sao Paulo	18054	9472	7441	18530	12227	0			
Sydney	7363	16989	11060	7792	12044	13376	0		
Singapore	2580	10852	8664	5307	5837	15999	6300	0	
New York	12967	5585	12833	10872	11028	7657	15988	15340	0

Figure S16. TSP instance and traveling path comparison. a The target problem instance solved in this paper. **b** The corresponding distance matrix between each city with the unit of km. The distance between each two cities is the shortest distance along the surface of earth and calculated by the coordinates of the city center. **c** The spin value matrix representation of the minimum-distance traveling path found by QPA. **d** Its corresponding traveling path in map. **e** The spin value matrix representation of the minimum-distance traveling path found by SA. **f** Its corresponding traveling path in map. **g** The spin value matrix representation of the minimum-distance traveling path found by DHNN. **h** its corresponding traveling path in map.

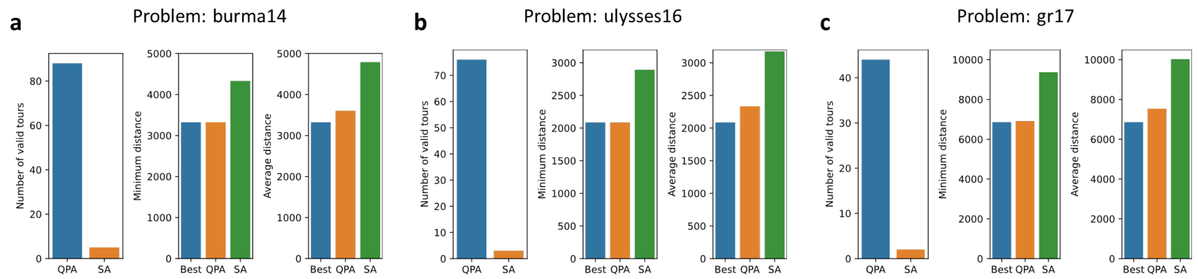

Figure S17. Simulation result of solving larger problems in TSPLIB [8]. a Result of solving burma14 problem with 14 cities **b** Result of solving ulysses16 problem with 16 cities **c** Result of solving gr17 problem with 17 cities. The Best in the plot represents the best-known solution provided by the library [8]. For each problem, 5000 iterations are conducted for both QPA and SA with 100 trials from random initial states.

Table S1. Energy and area estimation break down.

Module	Area/μm^2	Latency/ns	Energy/pJ
Array	137.21	<0.3	1.54
Drivers	275.56	0.1	0.34
S&H	3.16	-	0.02
MUX	101.80	0.008	0.20
ADC	14250	-	2.50
Sum	14767.73	-	4.60

Table S1 shows the estimated area and energy breakdown of one iteration in our memristor-based Ising solver. To ensure a fair comparison with mem-HNN [9], our estimations are based on 16 nm technology node. For the analog-to-digital converter (ADC), an 8-bit state-of-the-art successive-approximation-register (SAR) ADC design is used for estimation [10]. For the implementation of QPA, the same working frequency with mem-HNN of 500 Mhz (2 ns for each iteration) is used for benchmarking the time to solution. The power dissipation of this system is estimated by energy consumption for each iteration dividing the time needed for each iteration (4.60 pJ / 2 ns) and the result is 2.3 mW.

Note S1. Details of the estimation of benchmark comparison and comments on other techniques

In this paper, the performance of solving 100-node 50%-density unweighted Max-Cut problems was compared between our QPA implemented on memristor crossbar array and other six Ising machines based on different technologies. For our memristor-based QPA, the annealing time was set at 200 iterations, which results in a success probability of 16%. It takes 27 runs to reach a 99% success probability resulting in a time to solution of 5400 iterations. By using our current 180 nm technology node-based chip, the working frequency of which is 10Mhz (100 ns for each iteration) and the power dissipation of a single array in which is about 0.17 W. To run 5400 iterations, the calculated time to solution, energy to solution and solutions per second per Watt are 540 μs, 91.8 μJ and 1.09×10^4, respectively. Notably, the current hardware design is not optimized and is used for proof-of-concept purposes. To ensure a fair comparison with other technologies, we estimated our performance based on a 16 nm technology node (see in Table S1). The calculated time to solution, energy to solution and solutions per second per Watt based on our new estimation are 10.8 μs, 24.8 nJ and 4.03×10^7, respectively. For memristor-based Hopfield networks (mem-HNN), a hybrid updating scheme is utilized, which updates 10 nodes at a single iteration. To reach optimal time to solution, the annealing time is chosen as 50 updating cycles, where one updating cycle is equivalent to N/10 iterations (N is the problem size), resulting in the annealing time of 1 μs.1 μs. In addition, this hybrid updating method breaks the guaranteed convergence condition of simulated annealing and might lead to divergence, especially on denser problems with larger connectivity between nodes. More research is needed for the general adaptivity of this hybrid method. For phase-transition nano-oscillators (PTNO) based system, the fully analog property enables the low energy consumption. However, the experimental demonstration is limited to a problem size of N=8, which raises doubts about the effectiveness of the system on larger problems, especially considering of larger parasitic resistance and capacitance for larger problems. For ring oscillator (ROSC) based system, though the annealing time is ultra-fast compared to other techniques, its lack of annealing procedure results in a poor success probability and thus a relatively longer time-to-solution. As success probability of ROSC is not reported, an upper bound value of 1% is used for estimation, according to our previous experience on local-search algorithms. This results in the need of 459 runs to reach 99% success probability and the corresponding time to solution of 23 μs without considering the overhead brought by comparing such number of solutions. In addition, as ROSC system only supports sparse connection, to solve a dense problem, more spins are needed for embedding techniques which might further degrade the performance. For simulate bifurcation machine (SBM), the reported time to solution is for solving an all-to-all connected problem with both positive and negative couplings. However, since the difficulty of the problem does not change fundamentally, it is used for estimated comparison purposes here. Additionally, its energy consumption is estimated based on the power dissipation of field programmable gate array (FPGA), which is taken as 200W in this estimation. For coherent Ising machine (CIM), as the energy consumption is not reported, we have estimated a lower bound by considering only the energy consumption of the FPGA part (The power dissipation is assumed to be 200 W), without taking into account of the energy consumption of the optical part. For D-Wave 2000Q quantum systems, the energy consumption is limited by cryogenic cooling [9,11], and the speed of solving dense problems is limited by the spare connection [12].

Supplementary References

1 Leleu, T., Yamamoto, Y., Mcmahon, P. L. & Aihara, K. Destabilization of Local Minima in Analog Spin Systems by Correction of Amplitude Heterogeneity. *Phys. Rev. Lett.* **122** (2019). https://doi.org:10.1103/physrevlett.122.040607

2 Alizadeh, M., Fernández-Marqués, J., Lane, N. D. & Gal, Y. in *International conference on learning representations.*

3 Kingma, D. P. & Ba, J. Adam: A method for stochastic optimization. *arXiv preprint arXiv:1412.6980* (2014).

4 Le, H., Høier, R. K., Lin, C.-T. & Zach, C. in *Proceedings of the IEEE/CVF Conference on Computer Vision and Pattern Recognition.* 460-469.

5 Duchi, J., Hazan, E. & Singer, Y. Adaptive subgradient methods for online learning and stochastic optimization. *Journal of machine learning research* **12** (2011).

6 Zeiler, M. D. Adadelta: an adaptive learning rate method. *arXiv preprint arXiv:1212.5701* (2012).

7 Boixo, S. *et al.* Evidence for quantum annealing with more than one hundred qubits. *Nat. Phys.* **10**, 218-224 (2014). https://doi.org:10.1038/nphys2900

8 Reinelt, G. TSPLIB—A traveling salesman problem library. *ORSA journal on computing* **3**, 376-384 (1991).

9 Cai, F. *et al.* Power-efficient combinatorial optimization using intrinsic noise in memristor Hopfield neural networks. *Nat. Electron.* **3**, 409-418 (2020). https://doi.org:10.1038/s41928-020-0436-6

10 Liu, J., Hassanpourghadi, M. & Chen, M. S.-W. in *2022 IEEE International Solid- State Circuits Conference (ISSCC).* (IEEE).

11 Dutta, S. *et al.* An Ising Hamiltonian solver based on coupled stochastic phase-transition nano-oscillators. *Nat. Electron.* **4**, 502-512 (2021). https://doi.org:10.1038/s41928-021-00616-7

12 Hamerly, R. *et al.* Experimental investigation of performance differences between coherent Ising machines and a quantum annealer. *Sci. Adv.* **5**, eaau0823 (2019). https://doi.org:10.1126/sciadv.aau0823
